# Cross-cultural adaptation, reliability and validity of the Italian version of the craniofacial pain and disability inventory in patients with chronic temporomandibular joint disorders

**DOI:** 10.1186/s12903-019-0927-x

**Published:** 2019-11-12

**Authors:** Marco Monticone, Barbara Rocca, Paola Abelli, Simona Tecco, Tommaso Geri, Enrico Felice Gherlone, Deborah Luzzi, Marco Testa

**Affiliations:** 1 Department of Medical Sciences and Public Health, University of Cagliari, Cagliari, Italy; 2 Department of Neuroscience and Rehabilitation, Neurorehabilitation Unit, G. Brotzu Hospital, Cagliari, Italy; 3 Istituti Clinici Scientifici Maugeri, IRCCS, PRM Unit of Lissone, Lissone, Italy; 4 Istituti Clinici Scientifici Maugeri, IRCCS, PRM Unit of Montescano, Montescano, Italy; 5 Vita-Salute San Raffaele University, Dental Clinic, Milan, Italy; 6 Department of Neuroscience, Rehabilitation, Ophthalmology, Genetics, Maternal and Child Health, University of Genova - Campus of Savona, Savona, Italy

**Keywords:** Craniofacial pain disability inventory, Temporomandibular disorders, Cross-cultural adaptation, Psychometrics, Italian validation

## Abstract

**Background:**

To develop an Italian version of the Craniofacial Pain Disability Inventory (CFPDI-I) and investigate its psychometric abilities in patients with temporomandibular disorders (TMD).

**Methods:**

The CFPDI was translated following international standards. The psychometric analyses included reliability by internal consistency (Cronbach’s alpha) and test/retest stability (intraclass correlation coefficient, ICC); construct validity was investigated by matching (a priori hypotheses) the CFPDI-I with the Italian Neck Disability Index (NDI-I), a pain intensity numerical rating scale (NRS), the Italian Pain Catastrophising Scale (PCS-I), the Italian Tampa Scale of Kinesiophobia (TSK-I), and the Italian Migraine Disability Assessment Score Questionnaire (MIDAS) (Pearson’s correlation). Alpha was set at 0.05.

**Results:**

Two hundred and twelve patients with chronic TMD completed the tool. The questionnaire was internally consistent (α = 0.95) and its stability was good (ICCs = 0.91). As hypothesised, validity figures showed CFPDI-I strongly correlated with the NDI-I (*r* = 0.66, *p* < 0.05) and moderately correlated with the NRS (*r* = 0.48, *p* < 0.05), PCS (*r* = 0.37, *p* < 0.05), TSKI (*r* = 0.35, *p* < 0.05) and MIDAS (*r* = 0.47, *p* < 0.05). Similar estimates were shown by CFPDI-I subscales.

**Conclusions:**

The cross-culturally adapted version of the Craniofacial Pain and Disability Inventory (CFPDI-I) showed satisfactory psychometric properties that replicate those of the original version and, therefore, can be implemented in the clinical assessment of Italian people affected by TMD.

## Background

Clinicians are often involved in the diagnosis and conservative treatment of craniofacial and temporomandibular pain, inducing also a substantial impact on functional and psychosocial well-being [1, 2] Temporomandibular disorders (TMD) due to muscular, articular or mixed problems represent the second musculoskeletal complaints, after chronic low back pain, and result in craniofacial pain, headache and mood disorders [3].

It appeared therefore useful to quantify pain, disability and functional status of patients with craniofacial and TMD, based on a bio-psychosocial perspective. Indeed, the growing body of evidence coming from the biopsychosocial model suggests that, together with decreased physical activities, various psychosocial factors are important for the genesis and maintenance of chronic complaints and illness behaviors [4]. Appropriate assessment of physical and cognitive-behavioural factors is therefore increasingly advocated in research studies and clinical practice, and self-reported outcome measures seem to be the most applicable [5]. For this purpose, in 2014 the Craniofacial Pain and Disability Inventory (CFPDI, see Additional file 2 for Spanish version) was developed in patients with pain of the mandibular and craniofacial regions by means of item development and cognitive debriefing procedures. Exploratory analysis indicated a two-factor solution and the further assessment of its psychometric properties showed high internal consistency (Cronbach’s α: 0.88) and excellent test-retest reliability (ICC: 0.90). The CFPDI was closely correlated with a measure of neck disability (0.65), and moderately correlated with measures of pain intensity, catastrophising, kinesiophobia and headache impact (0.36–0.52). The instrument also proved to be easy to complete and requiring a relatively short time to administer [6]. The CFPDI was adapted and validated also in Brazilian and findings contributed to further exploring its reliability, construct and structural validity [7].

To the Authors’ knowledge, an Italian form of the CFPDI has never been created by means of full cross-cultural adaptation and psychometrically analysed. Full cross-cultural adaptations are crucial in order to guarantee the meanings of the original items are adequately captured in the target language and to subsequently allow psychometric testing (such structural and construct validity, internal consistency and reliability) of the original questionnaire, thus allowing comparisons between the results of investigations and original or other countries’ findings.

Therefore, Italian researchers and clinicians are restricted from interpreting the outputs available from this measure that, being developed from direct experience of patients with TMD and headaches, will help to capture the health condition of Italian people with craniofacial pain. Hence, the aim of this study was to made available a culturally translated and validated Italian form of the CFPDI for utilization in patients with chronic TMD.

## Methods

### Patients

The study gathered outpatients attending a physical medicine and rehabilitation unit and an affiliated centre between January 2015 and June 2017. The inclusion criteria were headache or facial pain attributable to TMD due to untreated muscular, articular or mixed complaints and a chronic condition defined as pain history of at least 6 months prior to the study, adult age, and confidence in Italian language; the exclusion criteria were systemic illness, psychiatric deficits, and recent cerebrovascular events or myocardial infarctions.

### CFPDI

It is a 21-item measure and patients give their answers using a 4-point Likert scale scoring from 1 (no problem) to 4 (maximum problem). The scale presents two factors, named *Pain and disability* (14 items) and *Jaw functional status* (7 items). The scores of the responses are added with higher scores indicating greater problems [6].

### Cross-cultural adaptation

The cross-cultural adaption was conducted in accordance with the American Association of Orthopaedic Surgeon Outcomes Committee protocol [8], taking into account also the rules shown in the ISPOR (International Society For Pharmacoeconomics and Outcomes) task force report “Principles of Good Practice for the Translation and Cultural Adaptation Process for Patient-Reported Outcomes (PRO) Measures” [9].

The following steps were conducted:

- *Step 1 Translation into another language (Italian)*: the items of the original CFPDI were adapted into Italian with the goal of maintaining the concepts of the original form, by means of clinically fitting terms. Two adaptations were made independently by two professional Italian translators qualified in this PRO area of study. The translators were given a plain description of the items in the CFPDI to fully understand their meaning. Keeping the language compatible with a comprehension age of 14 years, the differences in translations were solved by reconciliation between the translators; step 1 ended when a common translation was reached.
- *Step 2 Back-translation to the original language (Spanish)*: two bilingual translators whose mother tongue was Spanish back-translated independently the initial adaptation. The principal investigator (MM) reviewed the forms and stated that the new Italian measure reflected the same item content as the original scale and was conceptually comparable.
- *Step 3 Expert Committee*: to harmonize the above described procedure, the adaptations were sent to a bilingual committee of clinicians, methodologists and the translators chaired by the principal investigator, who explored the semantic, idiomatic and theoretical correspondence of the items and answers, to recognize any differences or errors. This phase ended when a pre-final version was shared.
- *Step 4 Test of the pre-final version*: the pre-final version was tested to assess the clarity and cognitive equivalence of the adaptation, highlight any items that may be unsuitable at theoretical level and discover any other problems that may induce uncertainty. This was done by cognitive interviews performed by an experienced psychologist (BR) who administered the scale to 30 patients with chronic TMD. The principal investigator and Expert Committee reviewed these findings with the goal of finding any change important to improve the Italian measure. (See Additional file 1 for final Italian version of CFPDI).

### Sample size

Ten patients per item were involved based on [10, 11].

### Scale properties

The original 21-item structure of the CFPDI was retained and used for all of the subsequent analyses [6].

#### Structural validity

A Confirmatory Factor Analysis (CFA) was performed to confirm the factor structure of the original version of the questionnaire [6], which suggested a 2 factors solution composed of, respectively, items 1, 2, 3, 4, 5, 6, 7, 8, 16, 17, 18, 19, 20, 21, and items 9, 10, 11, 12, 13, 14, 15. The model was fitted with lavaan version 0.6–3 [12] in R version 3.4.1 [13] . The estimation method was the Diagonal Weighted Least Square (DWLS) and the latent factors were standardized. The model fit was considered acceptable when the Comparative Fit Index (CFI) and the Tucker Lewis Index (TLI) were higher than 0.90 and 0.95 respectively [14], and the Root Mean Square Error of Approximation (RMSEA) was less than 0.05 and ideally close to 0. Modification indices and residuals were considered to explain the presence of local dependency among items.

#### Feasibility

The time needed to complete the measure was taken into account. The patients were asked about any problems while reading and responding the adapted scale, and the information were checked for missing or multiple answers.

#### Floor/ceiling effects

Descriptive statistics were calculated to recognize floor and/or ceiling effects, which were considered present when > 15% of the patients obtained the lowest or highest possible estimates, respectively [11].

#### Reliability

The reliability was investigated by internal consistency (Cronbach’s alpha, with estimates between 0.70 and 0.90 considered as acceptable) and test–retest stability (intraclass correlation coefficient: (ICC 2,1), with good and excellent reliability indicated by values of 0.70–0.85 and > 0.85, respectively) [10]. The absolute reliability between the two measurements was assessed with the 95% limits of agreement (LOA) and Bland and Altman plots [15] calculated in R [11] using the package BlandAltmanLeh version 0.3.1 [16] Stability was studied by giving the questionnaire to the same patient 7–10 days after the first administration.

#### Content validity

This was based on the patients’ answers to questions examining the goal of the CFPDI-I (*Question: “Do you think the aim of this scale is TMDs?”*), the target population (*“Do you think the items described may be associated to your pain?”*), relevance (“*Do you think these questions are important to investigating your TMDs?”*) and completeness (*“Do you think that these items exhaustively reveal your TMDs?”*). These hypotheses were considered acceptable if the percentage of positive answers was > 90% [11].

#### Construct validity

This was analysed by testing the hypotheses using outcome questionnaires as detailed below [9]. It was stated a priori there would be high and positive correlations between the CFPDI-I and a measure of disability, the Italian Neck Disability Index (NDI-I) [17], and moderate and positive correlations with a measure of pain intensity, the 0–10 numerical rating scale (NRS) [18]; a measure of catastrophising, the Italian Pain Catastrophizing Scale (PCS-I) [19]; a measure of kinesiophobia, the Italian Tampa Scale of Kinesiophobia (TSK-I) [20], and a measure of headache intensity, the Italian Migraine Disability Assessment Score Questionnaire (MIDAS) [21]. The following estimates of Pearson’s correlations were stated: *r* < 0.30 = low; 0.30 < *r* < 0.60 = moderate; *r* > 0.60 = high. Construct validity was considered good if > 75% of the hypotheses were established.

#### Sensitivity to change

This was estimated by the minimum detectable change (MDC) calculated by multiplying the standard error of the measurements (SEM) by the z-score associated with the desired level of confidence (95% in our study), and the square root of 2, which reflects the additional uncertainty introduced by using difference scores based on measurements made at test and retest. The SEM was calculated by the formula: SEM = SD[(1-R)^1/2^], where SD is the baseline standard deviation of the measurements, and R the test-retest reliability coefficient [11].

Statistics were made using the Italian SPSS 23.0 except for CFA and LOA, as described above.

## Results

### Patients

Two hundred thirty-three patients were invited to participate, of whom 212 accepted: 177 females (83.5%) and 35 males (16.5%) with a mean age of 47.7 ± 14.2 years (range 23–73). The median history of pain was 12 months (range 6–48). Thirteen patients (6.1%) presented myogenic pain, 56 (26.4%) showed arthrogenic pain and 143 (67.5%) presented disc disorders related pain. One hundred eighty-nine patients (89,15%) showed facial pain, and 150 patients (70,75%) had migraine. Their general characteristics are presented in Table 1.

**Table 1:** Sociodemographic characteristics of the population (*n* = 212)

Variable	Nos.	%
Marital status		
Unmarried	94	44.4
Married	118	55.6
Occupation		
Housewife	5	2.4
Pensioner	17	8.1
Student	27	12.7
Self-employed	66	31.1
Employee	97	45.7
Education		
Primary school	0	0
Middle school	20	9.4
High school	90	42.5
University	102	48.1
Smoking		
Yes	52	24.5
No	160	75.5
Drug use		
Antidepressants	41	19,3
Analgesics	62	29,2
Muscle relaxants	38	17,9
NSAIDs	57	26,9
None	14	6,7
Comorbidities (principal)		
Hypertension/heart diseases	33	15,6
NIDDM	23	10,8
Enteric disease	12	5,7
Liver disease	5	2,4
Other/None	139	65,5

*NSAIDs* non-steroidal anti-inflammatory drugs, *NIDDM* non-insulin-dependent diabetes mellitus

### Adaptation

The CFPDI was adapted into the Italian language by means of a process of forward/backward translation involving four professional translators. All the items were forward- and back-translated without difficulty, and no problems were raised during the check of the back-translations. The testing of the pre-final version shown no issues for patients during the completion of the questionnaire that warranted a consulting with a research assistant. The cognitive interviews confirmed the clarity and the cognitive correspondence of the adaptation without showing anything causing puzzlement. Therefore, the review by experts confirmed the suitability of the cross-cultural adaptation procedure, the content of the items, and their theoretical concepts. At last, the Principal Investigator and Expert Committee established the work done. The Additional file 1 displays the adapted Italian version of the questionnaire (CFPDI-I).

### Scale properties

#### Structural validity

**Fig. 1:**
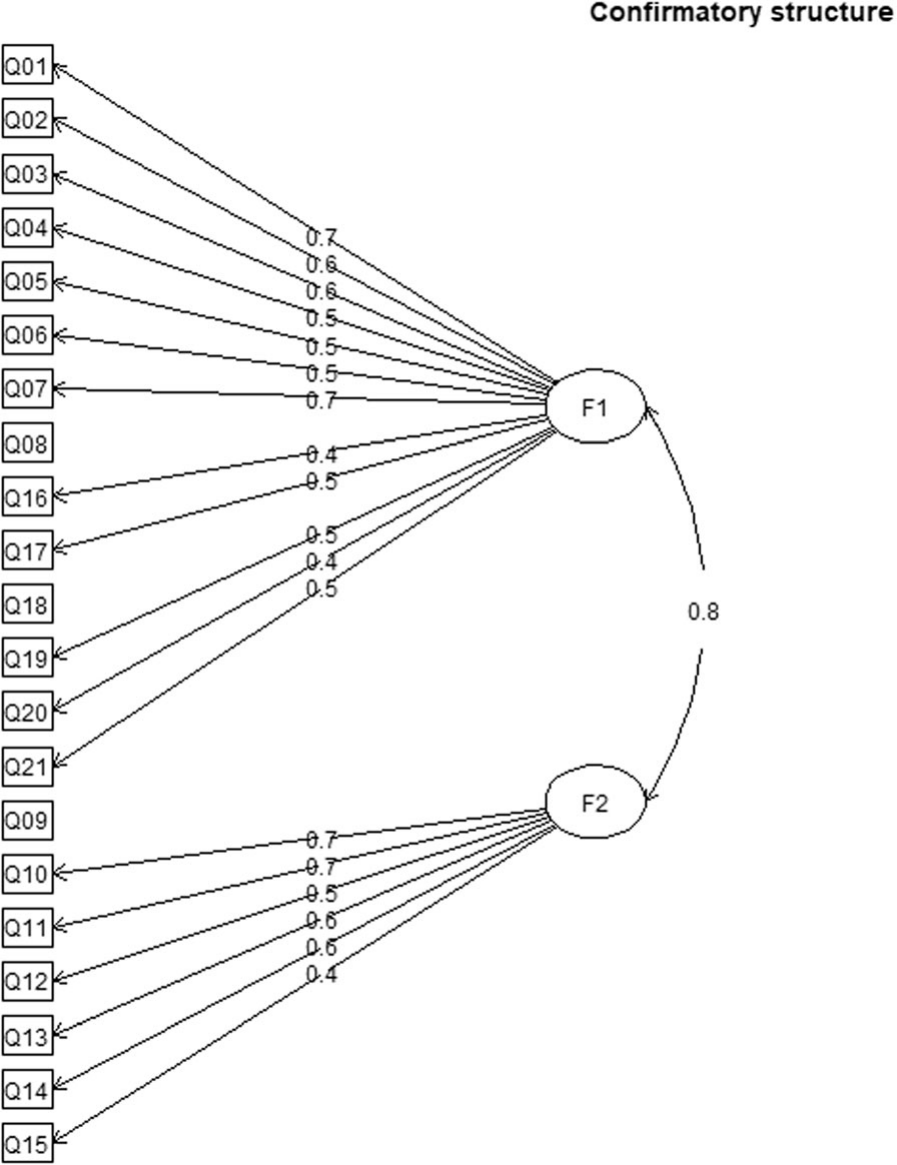
The diagram shows the two-factor structure of the CFPDI-I. Loadings are indicated on the arrows. Questions 8, 9 and 18 are not related to the factors because of their low loadings (see Results). F1, Pain and disability subscale; F2, Jaw functional status subscales. Q, Question

The result of CFA confirmed the 2-factors solution of the original version. The TLI was 0.95, the CFI was 0.96. The RMSEA was higher than the predefined threshold of 0.05 (RMSEA = 0.07, 90% CI 0.06–0.08, *p*-value = 0). The factor loadings reported in Table 2 were all satisfactory except for items 8, 9 and 18 that shown low loadings of the respective factor. The correlation between the 2 factors was 0.82 (*p* = 0), indicating the presence of a common variance components of both factors not explained by the model (Fig. 1). The high RMSEA value and between-factor correlation were explained by the presence of local dependency, especially for items 8 and 9, shown by high residuals (res) and modification indices (mi) for items 8 with factor 2 (mi =22.70), item 9 (res =0.32, mi =26.51), item 12 (res =0.15, mi =9.74), item 17 (res =0.19, mi =11.29), for item 9 with factor 1 (mi =11.98) and item 14 (res = .19, mi = 10.26), and for item 17 with item 9 (res =0.34, mi =18.75).

**Table 2:** Factor loadings from confirmatory factor analysis of the 2-factor solution suggested in the original version

Latent factor	Item	Estimate	SE	Z	*p*-value	Beta
Factor 1	1	0.67	0.03	25.25	0	0.76
	2	0.65	0.025	26.22	0	0.79
	3	0.60	0.02	25.36	0	0.67
	4	0.48	0.02	20.61	0	0.64
	5	0.54	0.03	20.70	0	0.70
	6	0.50	0.02	22.50	0	0.67
	7	0.74	0.03	25.58	0	0.74
	8	0.29	0.02	14.36	0	0.37
	16	0.38	0.02	17.22	0	0.43
	17	0.49	0.03	18.16	0	0.49
	18	0.26	0.02	11.12	0	0.33
	19	0.51	0.02	26.84	0	0.81
	20	0.44	0.02	20.88	0	0.67
	21	0.51	0.02	23.62	0	0.70
Factor 2	9	0.26	0.03	9.18	0	0.25
	10	0.68	0.03	20.79	0	0.74
	11	0.69	0.03	21.79	0	0.77
	12	0.54	0.03	15.67	0	0.54
	13	0.56	0.03	20.02	0	0.84
	14	0.57	0.03	16.54	0	0.67
	15	0.42	0.02	18.31	0	0.71

#### Feasibility

All of the questions were well received. The scale was answered in 5.20 ± 2.10 min; no missing responses or multiple answers in any of the questionnaires were highlighted, and there were no comprehension problems.

#### Floor/ceiling effects

No significant ceiling/floor effects were found in the total scale and the subscales.

#### Reliability

**Fig. 2:**
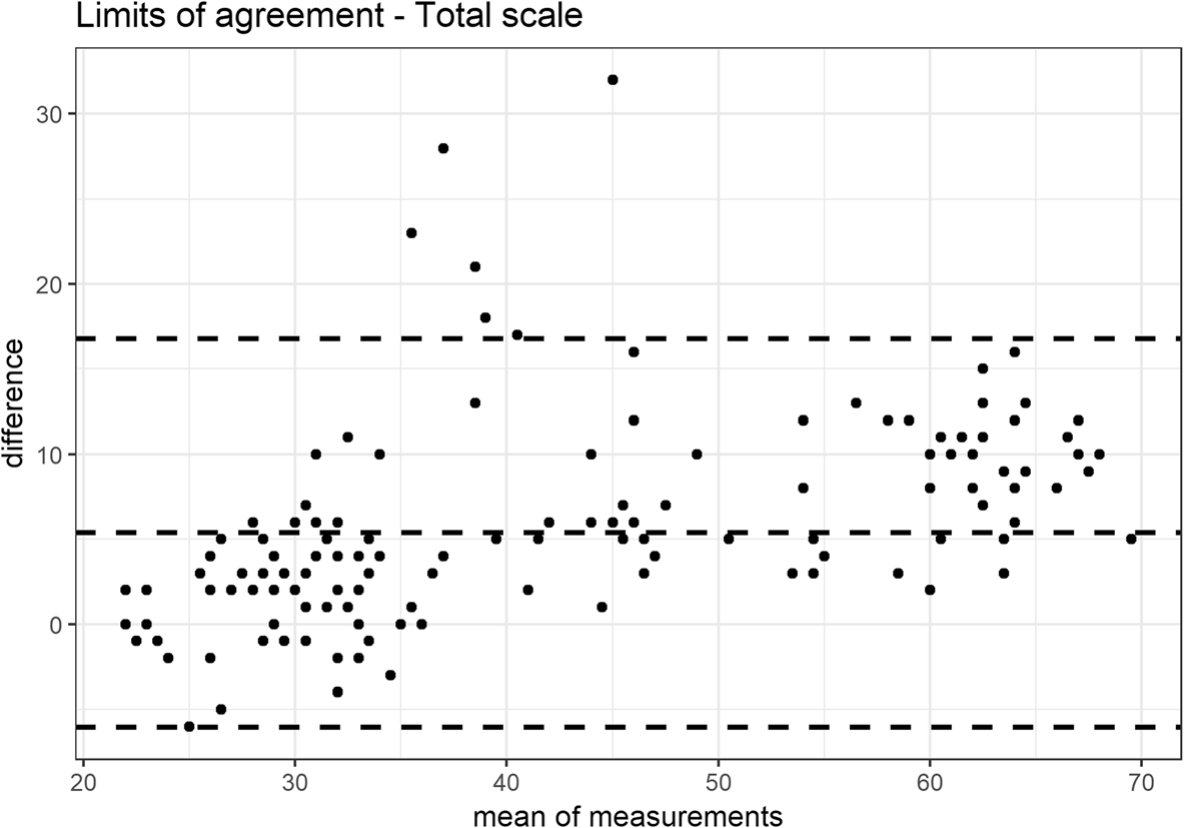
LOA of the total scale

**Fig. 3:**
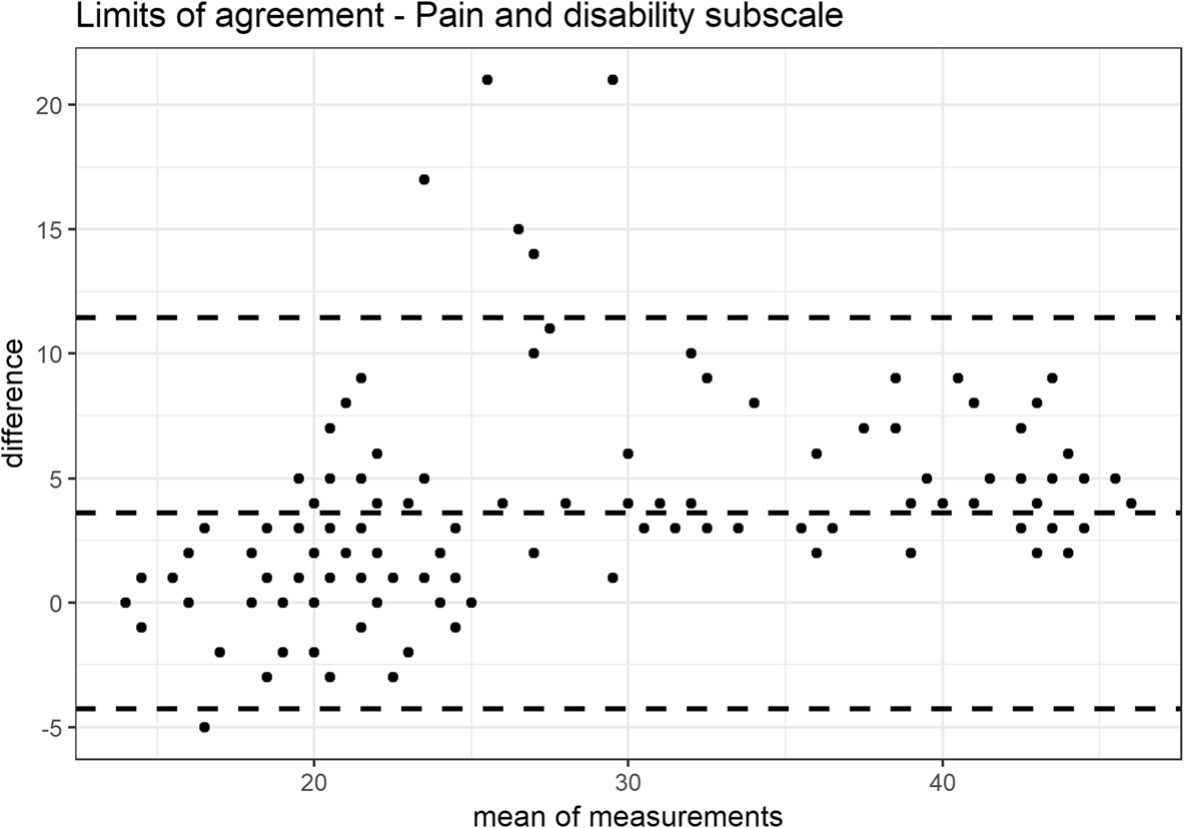
LOA of the pain and disability subscale

**Fig. 4:**
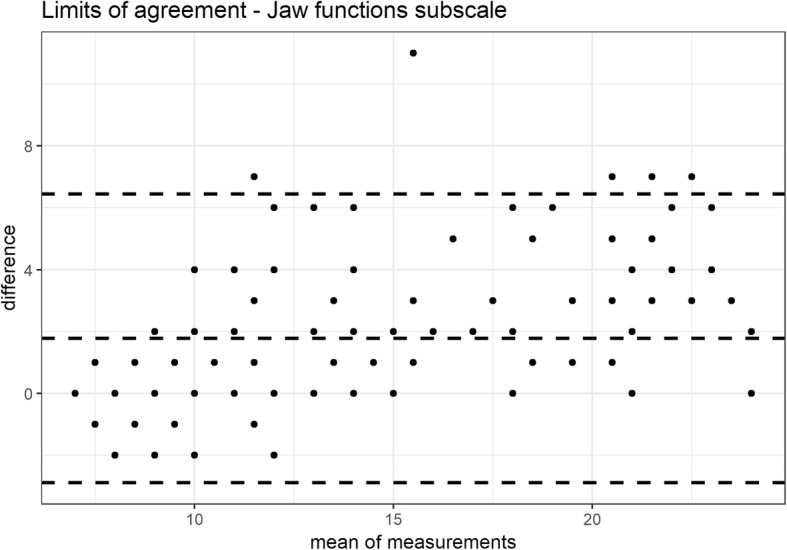
LOA of the jaw functions subscale

Cronbach’s α was higher than accepted. Test-retest stability was assessed in all the patients, and the scale presented excellent ICCs (see Table 3 for full results). For the absolute reliability, the LOA of the total scale ranged from − 6.0 to 16.8 points with a mean difference of 5.4 points (Fig. 2). The LOA of the pain and disability subscale ranged from − 4.2 to 11.44 points with a mean difference of 3.6 points (Fig. 3). The LOA of the jaw functions subscale ranged from − 2.9 to 6.4 points with a mean difference of 1.8 points (Fig. 4).

**Table 3:** Internal consistency, test-retest reliability and sensitivity to change of the Italian CF-PDI and its subscales

Scales	Internal consistency (α)	Test-retest (ICC 2,1 and 95% CI)	SEM	MDC
Total scale	0.95	0.86 (0.45–0.94)	5.5	15.2
Pain and disability	0.94	0.86 (0.47–0.94)	3.7	10.3
Jaw functions	0.96	0.85 (0.57–0.93)	2.1	5.7

*α* Cronbach’s alpha, *ICC* intraclass correlation coefficient, *CI* confidence interval, *SEM* Standard Error of Measurement, *MDC* Minimum Detectable Change

#### Content validity

The percentage of positive answers was always > 90%.

#### Construct validity

It was good because all of the a priori hypotheses were achieved (see Table 4 for full results).

**Table 4:** Construct validity of the Italian CF-PDI and its subscales

Variables and subscales	Total	Pain and disability	Jaw functions
NDI	0,66**	0,66**	0,62**
NRS	0,48**	0,48**	0,46**
PCS	0,37**	0,34**	0,43**
TSK	0,35**	0,32**	0,39**
MIDAS	0,47**	0,49**	0.43**

*NRS* numerical rating scale, *NDI* Neck Disability Index, *PCS* Pain Catastrophising Scale, *TSK* Tampa Scale of Kinesiophobia; *MIDAS* Migraine Disability Assessment Score Questionnaire

** *p* < 0.01

#### Sensitivity to change

The MDC was 15.2 for the total score, and 10.3 for *Pain and disability* and 5.7 for *Jaw functional status* subscales respectively, showing the smallest changes in score that are probable to reflect a true change instead of a measurement error. MDC and SEM values are reported in Table 3.

## Discussion

The process of translation of the CFPDI assured the concepts of the original items were satisfactorily captured by the idiomatic adaptation, leading to a valid measure of another culture’s conception of health that allows data comparability and cross-national studies. The measure resulted acceptable and effortlessly understood, and could be self-administered in about 5 min, rendering it appropriate for everyday clinical routine.

The result of the CFA confirmed the CFPDI had a structural validity similar to the original version as the TLI and CFI indices were satisfactory. The RMSEA value higher than the pre-defined threshold was due to the presence of local dependency of items 8 and 9. This is not surprising considering that a similar problem was experienced also by the authors of the original version, in which item 9 was not assigned to any of the 2 proposed factors but it was held in the questionnaire for theoretical reasons [6]. Furthermore, the exploratory factor analysis of the original version produced a scree plot with an inflexion point between 2 to 3 factors but with eigenvalues higher than 1. A 3-factors solution has been reported in the Brazilian Portuguese validation of the CFPDI after comparison with solution at 2 and 4 factors [7]. The main difference between the two-factor structure of the original version confirmed with the present study from the three-factor structure of Brazilian Portuguese version resides in modelling item 9 in a different factor. Therefore, the structural validity of the CFPDI still needs to be confirmed in future studies analysing the factor structure of the questionnaire.

Internal consistency was outside the range of the accepted values and had higher figures than the original versions (0.80–0.86) [6]. This aspect further confirms the high degree of inter-relatedness among the items, especially for items 8 and 9, which may refer to a similar construct that is not captured by the 2-factors solution. Indeed, the analysis of content of item 8, which refers to noise when moving the jaw, and item 9, which refers to feel the jaw getting out of place or getting stuck, may suggest that for Italian people the two items may be brought back to a construct referring to as “articular instability”. Also, Brazilian researchers investigated internal consistency which was slightly lower than our estimates (0.77) [7].

Test-retest stability was satisfactory and in line with the original findings (0.90) [6], confirming good repeatability over time in this population. Reliability over time was investigated also in the Brazilian sample and showed similar findings [7].

Correlation analyses showed that TMD was closely associated with neck disability, confirming its relationships with neck disorders [22–26] and suggesting the opportunity of testing TMD by means of the CFPDI-I in patients suffering from neck complaints and vice versa. Moderate associations were found with pain intensity, catastrophising and kinesiophobia: these findings are consistent with those of previous studies [27–29] suggesting that patients who persistently focus on TMD are more influenced by it in their thoughts, feelings and actions, more avoidant and generally show and report more pain-related behaviour and catastrophysing; hence, our findings suggest a role for multidisciplinary pain interventions including cognitive-behavioural therapy, and thus overcoming approaches typical of acute and subacute phases of musculoskeletal disorders [30]. Remarkably, the TSK-I used for validating the questionnaire was the Italian version for patients with low back pain [20]. There is also a TSK for TMD, but, unfortunately, there is no available Italian version [31]; it would be useful, as soon as an Italian validation of TSK-TMD will be available, to correlate it with CFPDI-I in order to complete the clinical information potentially made available by the CFPDI-I. When considering the other adapted version, the Brazilian study found a moderate correlation with neck disability (0.40) and strong correlations with TSK and PCS (0.68 and 0.69, respectively), confirming the above described relationships both with physical function as well as with psychological factors [7].

Further, moderate links were found between CFPDI-I and MIDAS, suggesting the importance to adequately evaluate the presence of migraine in patients with TMD [32–34]. Similar findings were also showed by the developers of the scale, confirming the associations also in the Italian context [6].

CFPDI-I showed to be less sensitive to change than the original version, that had an MDC of 7 points [6]. Based on the degree of repeatability, the SEM, MDC and LOA were low, and ensured it could highlight changes in the estimates exceeding the threshold of the measure noise. At a 95% confidence level, the minimum detectable change indicates that, if a subject shows a change of > 16 points after a given treatment, it would not be a measurement error. Another questionnaire to assess orofacial pain and oral health, the Oral Health Impact Profile-14 (IOHIP-14), is available in a validated Italian version [35]. Although it showed a high internal consistency (Cronbach’s α: 0.90), it demonstrated a fair test-retest reliability (ICC: 0.50–0.70) in comparison with the excellent test-retest reliability (0.86) of the CFPDI, value confirmed also by the Italian version [6].

This study shows some limits: it did not consider associations between TMD and physical tests as only self-reported measurements were utilised. Despite the sample size was calculated using previous recommendation [7], a greater sample size, without a high prevalence of females as in our sample, would have improved the generalizability of our results. Content validity was based on questions potentially preventing neutral answers, in part restraining the accuracy of our findings; thus, we recommend using open queries in the future. We did not introduce some of the most well-known instruments utilised to perform validation studies, such as the Short-Form Health Survey-36 (SF-36) items questionnaire, but researchers are advised to consider them in future studies to further investigate CFPDI-I properties. The TSK was not previously tested and validated in Italian patients suffering from TMD, limiting the interpretation of the correlations with the Italian CF-PDI. The MIDAS is not fully adequate to evaluate headache in general; however, we use it given the lack of other tools available in the Italian language. The use of drugs and the presence of comorbidities were high based on the sample included, probably affecting pain perception, disability, as well as pain beliefs; further analyses in samples suffering from lower levels of drugs use and comorbidities are therefore advised. Additional psychometric properties including the estimation of responsiveness and minimal important changes are also advised.

The sample enrolled in this study included patients with TMD. However, we do feel the characteristics and content of CFPDI items are not utterly related only to TMD and, therefore, the tool might be applicable to assess disability also in patients with other craniofacial pain (e.g. dental, neural or vascular origin).

## Conclusion

The Italian CFPDI shows good psychometric properties and its use can be suggested in TMD research also in Italy. This novel instrument is expected to help Italian researchers and clinicians in terms of diagnosis and treatment by highlighting key chronic pain beliefs and supplying further hints for successful approaches more based on cognitive-behavioural reconditioning within a bio-psychosocial model.

## Supplementary information

**Additional file 1.** The full questionnaire in its Italian form is presented.

**Additional file 2.** The full questionnaire in its original (Spanish) version is presented.

## Data Availability

All relevant data generated or analysed during this study are included in this published article. Raw data are available on request to authors.

## Abbreviations

CFPDI: Craniofacial Pain and Disability Inventory
CFPDI-I: Craniofacial Pain and Disability Inventory, Italian version
ICC: Intraclass Correlation Coefficient
IOHIP-14: Oral Health Impact Profile-14
ISPOR: International Society For Pharmacoeconomics and Outcomes
MDC: Minimum Detectable Change
MIDAS: Italian Migraine Disability Assessment Score Questionnaire
NDI-I: Italian Neck Disability Index
NRS: Numerical Rating Scale
PCS-I: Italian Pain Catastrophizing Scale
PRO: Patient-Reported Outcomes
SEM: Standard Error of the Measurements
SF-36: Short-Form Health Survey-36
TMD: Temporo-Mandibular Disorders
TSK-I: Italian Tampa Scale of Kinesiophobia
