# Supplementary material for: Cross-cultural adaptation, reliability and validity of the Italian version of the craniofacial pain and disability inventory in patients with chronic temporomandibular joint disorders
Source: BMC Oral Health. 2019 Nov 12;19:244. doi: 10.1186/s12903-019-0927-x (PMC6852744; doi:10.1186/s12903-019-0927-x)
Supplement: Supplementary file 1 — Additional file 1. The full questionnaire in its Italian form is presented. [file 12903_2019_927_MOESM1_ESM.doc]

**CFPDI - Italian version – 1.0**

**Questionario della disabilità e del dolore cranio-facciale**

Per favore, legga attentamente le istruzioni.

Questo questionario è stato creato per capire in che modo il dolore alla faccia, alla testa e alla mandibola influenzi le sue attività quotidiane. Per cortesia, risponda a tutte le domande possibili, segnando per ognuna solo la risposta che più si avvicina alla sua situazione. Anche se per qualche domanda esiste più di una opzione che si avvicina alla sua situazione, segni solo quella che rappresenta meglio il suo problema.

1. Prova dolore alla faccia?
   1. Non provo dolore.
   2. A volte provo dolore.
   3. Provo spesso dolore.
   4. Provo sempre dolore.
2. La sua qualità di vita è condizionata da questo dolore?
   1. Non è condizionata.
   2. E’ condizionata solo un po’.
   3. E’ condizionata molto.
   4. E’ condizionata moltissimo.
3. Intensità del dolore alla faccia.
   1. Non provo dolore.
   2. Provo un dolore leve.
   3. Provo un dolore moderato.
   4. Provo un dolore forte.
4. Il suo dolore le impedisce di avere relazioni affettuose come baci, abbracci, rapporti sessuali...?
   1. Non ho nessuna difficoltà ad avere queste relazioni affettuose.
   2. Posso averle, ma con un dolore lieve alla faccia e/o alla mandibola.
   3. Posso averle, ma con un dolore moderato alla faccia e/o alla mandibola.
   4. Non le ho a causa del dolore grave che provo.
5. Prova dolore quando ride?
   1. Non provo dolore.
   2. Provo un dolore leve.
   3. Provo un dolore moderato.
   4. Provo un dolore forte.
6. Il suo dolore le impedisce di sorridere, parlare o masticare?
   1. Posso compiere queste azioni senza alcun problema.
   2. A volte le evito per il dolore che mi provocano.
   3. Molto spesso le evito per il dolore che mi provocano.
   4. Le evito sempre per il dolore che mi provocano.
7. Prova dolore alla mandibola?
   1. Non provo dolore.
   2. Provo dolore solo quando la muovo.
   3. Anche se non la muovo, a volte provo dolore.
   4. Provo un dolore continuo, indipendentemente da ciò che faccio.
8. Sente qualche rumore quando muove la mandibola?
   1. Non sento nessun rumore.
   2. Sento rumore solo in qualche movimento.
   3. Sento rumore e provo anche dolore nella maggior parte dei movimenti.
   4. Sento rumore e provo anche dolore in ogni movimento.
9. Sente che la sua mandibola esce di posto o si blocca?
   1. Non sento niente di strano.
   2. A volte sento che la mia mandibola esce di posto o si blocca.
   3. Sento spesso che la mia mandibola esce di posto o si blocca.
   4. Sento sempre che la mia mandibola esce di posto o si blocca.
10. Intensità del dolore quando si mastica.
    1. Non provo dolore.
    2. Provo un dolore lieve.
    3. Provo un dolore moderato.
    4. Provo un dolore forte.
11. Prova stanchezza alla mandibola quando parla o mangia?
    1. Non provo stanchezza.
    2. Provo una lieve stanchezza.
    3. Provo una stanchezza moderata.
    4. Provo una stanchezza intensa.
12. Ha difficoltà ad aprire la bocca?
    1. Non ho difficoltà ad aprire la bocca.
    2. Ho lieve difficoltà ad aprire la bocca.
    3. Ho difficoltà moderata ad aprire la bocca.
    4. Ho difficoltà intensa ad aprire la bocca.
13. Intensità del dolore a parlare.
    1. Non provo dolore.
    2. Provo un dolore lieve.
    3. Provo un dolore moderato.
    4. Provo un dolore forte.
14. Ha paura a muovere la mandibola?
    1. Non ho paura a muovere la mandibola.
    2. A volte evito di fare alcuni movimenti della mandibola, perché ho paura che il mio problema possa peggiorare.
    3. Spesso evito di fare alcuni movimenti della mandibola, perché ho paura che il mio problema possa peggiorare.
    4. Faccio solo i movimenti strettamente necessari, perché ho paura che il mio problema possa peggiorare.
15. Alimentazione.
    1. Posso mangiare qualsiasi alimento.
    2. Non posso mangiare alcuni alimenti di consistenza dura.
    3. Posso mangiare solo alimenti morbidi.
    4. Assumo solo alimenti liquidi.
16. Con che frequenza prova dolore al collo?
    1. Non provo dolore al collo.
    2. A volte provo dolore al collo.
    3. Spesso provo dolore al collo.
    4. Provo continuamente dolore al collo.
17. Con che frequenza prova mal di testa?
    1. Non provo mal di testa.
    2. A volte provo mal di testa.
    3. Spesso provo mal di testa.
    4. Provo continuamente mal di testa.
18. Con che frequenza prova mal d’orecchi?
    1. Non provo mal d’orecchi.
    2. A volte provo mal d’orecchi.
    3. Spesso provo mal d’orecchi.
    4. Provo continuamente mal d’orecchi.
19. Cosa prova quando tocca la zona che le fa male?
    1. Se esercito una pressione lieve con le dita sulla faccia, non mi fa male.
    2. Se esercito una pressione lieve con le dita sulla faccia, mi fa un po’ male.
    3. Se esercito una pressione lieve con le dita sulla faccia mi fa molto male.
    4. Non posso toccarmi la faccia, perché solo sfiorarla mi fa molto male.
20. Il dolore turba il suo sonno?
    1. Il dolore non turba il mio sonno.
    2. A volte il dolore non mi permette di prendere sonno.
    3. Spesso il dolore non mi permette di prendere sonno.
    4. Non posso dormire perché il dolore non mi permette di addormentarmi.
21. Il dolore interferisce con la sua attività lavorativa?
    1. Non interferisce con la mia attività lavorativa.
    2. A volte interferisce con la mia attività lavorativa.
    3. Spesso interferisce con la mia attività lavorativa.
    4. Interferisce sempre con la mia attività lavorativa.

Punteggio totale: …../84.
