# Supplementary material for: Cross-cultural adaptation, reliability and validity of the Italian version of the craniofacial pain and disability inventory in patients with chronic temporomandibular joint disorders
Source: BMC Oral Health. 2019 Nov 12;19:244. doi: 10.1186/s12903-019-0927-x (PMC6852744; doi:10.1186/s12903-019-0927-x)
Supplement: Supplementary file 2 — Additional file 2. The full questionnaire in its original (Spanish) version is presented. [file 12903_2019_927_MOESM2_ESM.pdf]

Inventario de Discapacidad y Dolor Craneofacial (IDD-CF)

Por favor, lea atentamente las instrucciones:

Este cuestionario se ha diseñado para obtener información de cómo le afecta a su vida diaria el dolor sobre la cara, la cabeza y la mandíbula. Por favor, rellene todas las preguntas posibles y marque en cada una *SÓLO LA RESPUESTA QUE MÁS SE APROXIME A SU CASO*. Aunque observe que en alguna pregunta existe más de una opción que refleje su caso, marque sólo la opción que represente mejor su problema.

1. ¿Presenta dolor en la cara?

○ No tengo dolor.
○ En algunas ocasiones tengo dolor.
○ Frecuentemente tengo dolor.
○ Siempre tengo dolor.

2. ¿Se ha visto afectada su calidad de vida por esta dolencia?

○ No se ha visto afectada.
○ Se ha visto algo afectada.
○ Se ha visto muy afectada.
○ Se ha visto severamente afectada.

3. Intensidad de dolor en la cara.

○ No siento dolor.
○ Siento un dolor leve.
○ Siento un dolor moderado.
○ Siento un dolor severo.

4. Le incapacita su dolor a la hora de tener relaciones afectuosas del tipo: besos, abrazos, relaciones sexuales…

○ No tengo ninguna incapacidad a la hora de mantener relaciones afectuosas.
○ Puedo mantenerlas, pero con un dolor leve en la cara y/o mandíbula.
○ Puedo mantenerlas, pero con un dolor moderado en la cara y/o mandíbula.
○ Me abstengo de mantenerlas debido al severo dolor que siento.

5. ¿Tiene dolor al reír?

○ No siento dolor.
○ Siento un dolor leve.
○ Siento un dolor moderado.
○ Siento un dolor severo.

6. ¿Su dolencia hace que evite el sonreír, hablar o masticar?

○ Puedo realizar estos gestos o funciones sin ningún problema.
○ En algunas ocasiones los evito por el dolor que me produce.
○ Frecuentemente los evito por el dolor que me produce.
○ Siempre los evito por el dolor que me produce.

7. ¿Tiene dolor en la mandíbula?

○ No tengo dolor.
○ Solo tengo dolor cuando la muevo.
○ Aunque no la muevo en ocasiones tengo dolor.
○ El dolor es constante independientemente de la actividad.

8. ¿Escucha algún ruido al mover la mandíbula?

○ No escucho ningún ruido.
○ En algún movimiento noto un ruido.
○ En la mayoría de los movimientos noto ruidos y además dolor.
○ En todos los movimientos noto ruido y además dolor.

9. ¿Nota que su mandíbula se le sale o se le traba?

○ No noto ninguna sensación fuera de lo común.
○ En algunas ocasiones siento que la mandíbula se me sale o se me traba.
○ Frecuentemente siento que la mandíbula se me sale o se me traba.
○ Siempre siento que la mandíbula se me sale o se me traba.

10. Intensidad de dolor al masticar…

○ No siento dolor.
○ Siento un dolor leve.
○ Siento un dolor moderado.
○ Siento un dolor severo.

11. **¿Siente cansancio en la mandíbula, al hablar o al comer?**

- ○ No tengo cansancio.
- ○ Siento un cansancio leve.
- ○ Siento un cansancio moderado.
- ○ Siento un cansancio severo.

12. **¿Tiene dificultad para abrir la boca?**

- ○ No tengo dificultad para abrir.
- ○ Tengo una leve dificultad para abrir.
- ○ Tengo una moderada dificultad para abrir.
- ○ Tengo una severa dificultad para abrir.

13. **Intensidad de dolor al hablar...**

- ○ No siento dolor.
- ○ Siento un dolor leve.
- ○ Siento un dolor moderado.
- ○ Siento un dolor severo.

14. **¿Tiene miedo de mover la mandíbula?**

- ○ No tengo miedo de mover la mandíbula.
- ○ En algunas ocasiones evito realizar algunos movimientos de la mandíbula por miedo a que mi problema empeore.
- ○ Frecuentemente evito realizar algunos movimientos de la mandíbula por miedo a que mi problema empeore.
- ○ Solo realizo los movimientos estrictamente necesarios por miedo a que mi problema empeore.

15. **Alimentación**

- ○ Puedo comer cualquier alimento.
- ○ Algunos alimentos duros no los puedo comer.
- ○ Solo puedo comer alimentos blandos.
- ○ Solo me alimento con líquidos.

16. **¿Con qué frecuencia tiene dolor en el cuello?**

- ○ No tengo dolor en el cuello.
- ○ En algunas ocasiones tengo dolor de cuello.
- ○ Frecuentemente tengo dolor de cuello.
- ○ Siempre tengo dolor de cuello.

17. **¿Con qué frecuencia tiene dolor de cabeza?**

- ○ No tengo dolor de cabeza.
- ○ En algunas ocasiones tengo dolor de cabeza.
- ○ Frecuentemente tengo dolor de cabeza.
- ○ Siempre tengo dolor de cabeza

18. **¿Con qué frecuencia tiene dolor de oído?**

- ○ No tengo dolor de oído.
- ○ En algunas ocasiones tengo dolor de oído.
- ○ Frecuentemente tengo dolor de oído.
- ○ Siempre tengo dolor de oído.

19. **¿Qué siente al tocarse la zona dolorosa?**

- ○ Si realizo una leve presión con los dedos sobre la cara no me produce dolor.
- ○ Si realizo una leve presión con los dedos sobre la cara me produce un dolor leve.
- ○ Si realizo una leve presión con los dedos sobre la cara me produce un dolor intenso.
- ○ No puedo tocarme la cara porque solo el roce me produce mucho dolor.

20. **¿Su dolor le altera el sueño?**

- ○ El dolor no me altera el sueño.
- ○ En ocasiones el dolor me impide conciliar el sueño.
- ○ Frecuentemente el dolor me impide conciliar el sueño.
- ○ No puedo dormir debido a que el dolor me impide conciliar el sueño.

21. **¿El dolor le interfiere a la hora de desempeñar su actividad laboral?**

- ○ No me interfiere en mi actividad laboral.
- ○ En algunas ocasiones interfiere en mi actividad laboral.
- ○ Frecuentemente interfiere en mi actividad laboral.
- ○ Siempre interfiere en mi actividad laboral.
